# Employing a Qualitative Description Approach in Health Care Research

**DOI:** 10.1177/2333393617742282

**Published:** 2017-11-24

**Authors:** Carmel Bradshaw, Sandra Atkinson, Owen Doody

**Affiliations:** 1 University of Limerick, Limerick, Ireland

**Keywords:** qualitative description, philosophy, epistemology, ontology, methodology and methods

## Abstract

A qualitative description design is particularly relevant where information is required directly from those experiencing the phenomenon under investigation and where time and resources are limited. Nurses and midwives often have clinical questions suitable to a qualitative approach but little time to develop an exhaustive comprehension of qualitative methodological approaches. Qualitative description research is sometimes considered a less sophisticated approach for epistemological reasons. Another challenge when considering qualitative description design is differentiating qualitative description from other qualitative approaches. This article provides a systematic and robust journey through the philosophical, ontological, and epistemological perspectives, which evidences the purpose of qualitative description research. Methods and rigor issues underpinning qualitative description research are also appraised to provide the researcher with a systematic approach to conduct research utilizing this approach. The key attributes and value of qualitative description research in the health care professions will be highlighted with the aim of extending its usage.

## Introduction

There is a myriad of qualitative approaches to research. Yet, the researcher may be confronted with a question or a topic that belongs within the qualitative paradigm but does not correspond neatly with approaches that are well documented and clearly delineated. Within the literature, various terms have been used to describe research that does not fit within a traditional qualitative approach. Thorne, Kirkham, and MacDonald-Emes (1997) define “interpretive description” as a “noncategorical” qualitative research approach (p. 169). Merriam (1998) refers to this type of research as “basic or generic qualitative research” (p. 20) and Sandelowski (2000, p. 335, 2010) explores what she calls “basic or fundamental qualitative description.” Exploratory research is the umbrella term used by Brink and Wood (2001) to describe all description qualitative research and suggest it “is a Level 1 research endeavor” (p. 85), and Savin-Baden and Howell Major (2013) refer to a pragmatic qualitative approach. This interchangeable use of terms creates ambiguity and confusion in relation to qualitative description research as a methodology in its own right. Reference to “interpretive” as described by Thorne et al. (1997) can cause confusion with phenomenology, for example, and Savin-Baden and Howell Major’s (2013) use of a “pragmatic qualitative approach” might suggest that if all else fails, the researcher should adopt a pragmatic approach.

A clear identification of qualitative description research is required, one that best captures what it does to aid researchers in determining which approach best suits the question or phenomenon which has been identified for exploration. Qualitative description research studies are those that represent the characteristics of qualitative research rather than focusing on culture as does ethnography, the lived experience as in phenomenology or the building of theory as with grounded theory. Qualitative description research studies are those that seek to discover and understand a phenomenon, a process, or the perspectives and worldviews of the people involved (Caelli, Ray, & Mill, 2003; Merriam, 1998). As a methodology, qualitative description research studies have gained popularity in recent years within nursing and midwifery, and Polit and Beck (2014) identified they accounted for more than half of qualitative studies. The use of a qualitative description approach is particularly relevant where information is required directly from those experiencing the phenomenon under investigation, where time and resources are limited and perhaps as part of a mixed methods approach (Neergaard, Oleson, Anderson, & Sondergaard, 2009).

## Philosophical Assumptions

Philosophical perspectives dictate what constitutes knowledge and how phenomena should be studied (Weaver & Olson, 2006), thus assisting researchers to refine and specify the types of evidence necessary, how it should be collected, and how it should be interpreted and used. Qualitative description research lies within the naturalistic approach, which creates an understanding of a phenomenon through accessing the meanings participants ascribe to them. The study of phenomena in their natural context is central, along with the acceptance that researchers cannot evade affecting the phenomenon under investigation. A value neutral position can never be adopted by the naturalistic researcher and their philosophy is central to the phenomena under investigation (Parahoo, 2014). There can be no reality without understanding language and acknowledging the researcher’s preconceptions, and only through subjective interpretation can this reality be truly uncovered. The philosophical assumptions identified by the authors of this article are identified in Table 1. These can guide the researcher in their ontology and epistemology assumptions, which directs subsequent methodology providing a framework to accomplish a study using a qualitative description approach.

**Table 1.** Philosophical Underpinnings of Qualitative Description Approach.

● An inductive process (describes a picture of the phenomenon that is being studied, and can add to knowledge and develop a conceptual and/or theoretical framework). ● Is subjective (each person has their own perspective and each perspective counts). Recognizes the subjectivity of the experience of not only the participant but also the researcher ● Designed to develop an understanding and describe phenomenon (not to provide evidence for existing theoretical construction). ● Researcher is active in the research process (researcher becomes part of the phenomenon being studied as they talk directly to participants and/or observe their behaviors). ● An emic stance (an insider view which takes the perspectives and words of research participants as its starting point) but is influenced by the researcher not only because of subjectivity but also when a degree of interpretation occurs. ● Conducted in the natural setting (data collected in the natural setting of the participants who experience the phenomenon).

Source. Developed by the authors. Sullivan-Bolyai, Bova, and Harper (2005) also make a compelling argument for the use of qualitative description in health care research because of its ability to provide clear information on how to improve practice. In addition, other qualitative approaches may not be appropriate for the issue requiring exploration or investigation. Furthermore, the findings emanating from such studies can often create a platform for more extensive and focused work on the topic. The misconception that qualitative description research is less theoretical or methodologically sound is unmerited as evidenced by Sandelowski (2000, 2010), Sullivan-Bolyai et al. (2005), and Neergaard et al. (2009). This article addresses the philosophical, ontological, epistemological methods and rigor underpinning qualitative description methodology and aims to provide the researcher with a systematic approach to conducting research utilizing a qualitative description design.

## Ontological Assumptions

Ontology is the study of being (Crotty, 1998) and is concerned with what constitutes reality, what the real world is, and what can be known about it (Denzin & Lincoln, 2011). The ontological position of naturalistic research is relativism, which holds the view that reality is subjective and varies from person to person (Parahoo, 2014) and this is evident in the reporting of findings from qualitative description research. Realities are influenced by senses and emerge when consciousness engages with objects, which already have meaning for the individual (Crotty, 1998, p. 43). What follows is that there are many realities, and no one reality can exist as individuals ascribe their own interpretation and meaning to the phenomenon. In addition, the use of language actively shapes and molds our reality (Frowe, 2001). Thus, reality is constructed through the interaction between language and aspects of an independent world where people’s description of a phenomenon can be seen as either a proxy or literal description or a combination of both. Qualitative description research strives for in-depth understanding but with emphasis first on literal description (Sandelowski, 2010) and then on the understanding of human phenomena through analysis and interpretation of meaning people ascribe to events.

## Epistemological Assumptions

Epistemological assumptions relate to how knowledge can be created, developed, and communicated, in other words, what it means to know and involves asking what is the nature of the relationship between the would-be knower and what can be known (Denzin & Lincoln, 2011). The epistemological position of qualitative research is subjectivism, which is based on real-world phenomena; the world does not exist independently of our knowledge of it (Grix, 2004). Subjectivism accepts the reality of all objects, relies entirely on an individual’s subjective awareness of it, and stresses the role and contribution the researcher plays, and this is congruent with the qualitative description approach to research.

The qualitative description approach accepts that many interpretations of reality exist and that what is offered is a subjective interpretation strengthened and supported by reference to verbatim quotations from participants. Knowledge of reality from a naturalistic perspective as is the case in qualitative description research is socially constructed not only by the participants obviously but also by the researchers, and it is therefore recognized that an objective reality cannot be discovered or replicated by others.

## Methodological Assumptions

Methodological assumptions consider how researchers approach finding out what they believe can be known (Denzin & Lincoln, 2011), finding the best fit to the phenomena under investigation in a pragmatic manner. Within qualitative description, the outcome is to describe the phenomenon literally as a starting point and its methodological orientation may be drawn from a range of theorists, for example, Sandelowski (2000). Qualitative description design then moves beyond the literal description of the data and attempts to interpret the findings without moving too far from that literal description. Stating one’s theoretical orientation will help readers understand how research methods are decided, for example, data collection, data analysis, interpretation, findings presentation, and rigor. Within the qualitative description approach, the phenomenon of interest is explored with participants in a particular situation and from a particular conceptual framework (Parse, 2001) with the research question related to the meaning of the experience. The participants are a purposive or purposeful sample who have the requisite knowledge and experience of the phenomena being researched. The interactions of a given social unit are investigated and the “participant group is selected from the population the researcher wishes to engage in the study” (Parse, 2001, p. 59). The descriptions obtained from participants are then analyzed and synthesized from the perspective of the chosen framework. Researchers aiming to use a qualitative description approach need to address from the outset (as indeed do all researchers regardless of approach) their theoretical positioning, congruence between methodology and methods, strategies to establish rigor, and the analytic lens through which data analysis is conducted.

The goal of qualitative description research is not “discovery” as is the case in grounded theory, not to “explain” or “seeking to understand” as with ethnography, not to “explore a process” as is a case study or “describe the experiences” as is expected in phenomenology (Doody & Bailey, 2016). Qualitative description research seeks instead to provide a rich description of the experience depicted in easily understood language (Sullivan-Bolyai et al., 2005). The researcher seeks to discover and understand a phenomenon, a process, or the perspectives and worldviews of the people involved (Caelli et al., 2003). A qualitative description approach, therefore, offers the opportunity to gather rich descriptions about a phenomenon which little may be known about. Within the process, the researcher strives to stay close to the “surface of the data and events” (Sandelowski, 2000, p. 336), where the experience is described from the viewpoint of the participants (Sullivan-Bolyai et al., 2005).

The goal of the researcher is to provide an account of the “experiences, events and process that most people (researchers and participants) would agree are accurate” (Sullivan-Bolyai et al., 2005, p. 128). The focus on producing rich description about the phenomenon from those who have the experience offers a unique opportunity to gain inside or emic knowledge and learn how they see their world.

Two main elements constant with qualitative description studies in health care research are learning from the participants and their descriptions, and second, using this knowledge to influence interventions (Sullivan-Bolyai et al., 2005). Therefore, a fundamental qualitative description design is valuable in its own right. Qualitative description studies are typically directed toward discovering the who, what, where, and why of events or experiences (Neergaard et al., 2009). A qualitative descriptive approach does not require the researcher to move as far from the data and does not require a highly abstract rendering of data compared with other qualitative designs (Lambert & Lambert, 2012) but of course does result in some interpretation. The findings from these studies can often be of special relevance to practitioners and policy makers (Sandelowski, 2000).

## Methods Assumptions

Methods refer to the tools, techniques, or procedures used to gather and interpret evidence. Researchers employing a qualitative description approach must clearly articulate their disciplinary connection, what brought them to the question, and the assumptions they make about the topic of interest. The tools used to collect and analyze the data must be congruent with the philosophical, epistemological, and ontological assumptions underpinning the research (van Manen, 1998). In their results, researchers must demonstrate congruence between the questions posed and the approach employed. Some methods have their origins in a particular methodology, for example, constant comparative methods as in grounded theory (Glaser & Strauss, 1967). However, a variety of methods can be utilized in qualitative description research as long as they are congruent with the research question and the purpose of the research, and contribute to the rigor of the research. In research methods researchers can address: ethics, sampling, collecting and analyzing rich data (Polit & Beck, 2014; Sandelowski, 2000); and extensive interaction with participants (Streubert & Carpenter, 2011). A flexible plan of inquiry that is responsive to real-world contexts (Patterson & Morin, 2012), naturalistic study methods (Holloway, 2005; Sandelowski, 2000), and rigor can also be included in research methods.

### Sampling and Sample Size

It is essential that the sampling techniques selected within a research study are reflective of the research design and research question. The sampling process best able to achieve this within qualitative studies and in particular qualitative description designs is a nonprobability technique of convenience or purposive sampling (Parahoo, 2014). Convenience sampling allows the researcher to select participants who are readily accessible or available. Likewise, purposive sampling avails of accessible participants, but it provides the additional advantage of facilitating the selection of participants whose qualities or experiences are required for the study.

The size of the sample has generated discussion among qualitative researchers. Qualitative samples tend to be small because of the emphasis on intensive contact with participants and the findings are not expected to be generalizable. The principle of “data saturation” has become an accepted standard to determine sample size within qualitative designs. However, the difficulties and challenges regarding the concept of “data saturation” have recently been debated (Fusch & Ness, 2015; Malterud, Siersma, & Guassora, 2015). The concept originated from “theoretical saturation,” an element of constant comparative method, which is a specific component of grounded theory methodology (Glaser & Strauss, 1967). However, in other qualitative research designs, the concept of “data saturation” has a number of definitions and is rarely made explicit within research studies (O’Reilly & Parker, 2013). Data saturation can be considered to apply to the point where no new information emerges from the study participants during data collection (Coyne, 1997), when the ability to obtain new information has been attained and when additional coding is no longer feasible (Guest, Bunce, & Johnson, 2006) or when enough information is gathered to replicate the study (Walker, 2012). However, data saturation is often referred to in a pragmatic manner to signal the end of data collection. The concept of data saturation is also contested within other qualitative research designs such as phenomenology, and in particular, hermeneutic phenomenology (Ironside, 2006) and Interpretative Phenomenological Analysis (Smith, Flowers, & Larkin, 2009). These research designs stress the uniqueness of each individual’s experience (mirroring the philosophy of qualitative description design) and therefore argue that data saturation can never truly be reached (Ironside, 2006). LoBiondo-Wood and Haber (2014) concur and suggest that there is no fixed rule to establish the most appropriate sample size in qualitative research, instead a number of factors should be considered. These include careful consideration of the research design, sampling procedure, and the relative frequency of the phenomena being researched. Therefore, according to Fawcett and Garity (2009), an adequate sample size is one that sufficiently answers the research question, the goal being to obtain cases deemed rich in information. Therefore, consideration can be given to include tentative sample sizes in any proposal delineating a qualitative description approach. It is evident that regardless of the strategies engaged in sampling and subsequently sample size, all research studies are required to defend their sampling strategies and provide clarity as to how sample size was determined to meet the objectives of the study.

### Ethics

Cluett and Bluff (2006) emphasize a researcher’s responsibility to address ethical principles relevant to their study to demonstrate “*professional, legal and social accountability*” (p. 199). There are a number of ethical principles that a researcher must address prior to and throughout the research process to safe guard the participant and uphold the integrity of the study. In particular, participants’ confidentiality and anonymity can be compromised as data collection methods, for example, face-to-face interviews, which are more intimate, are often used in qualitative description designs due to the open-ended nature of data collection. The more information researchers give when constructing a rich description, the greater the danger of participant identification. Researchers may have to mask contextualization to some extent to protect participants’ identities, while still ensuring that what is reported is verbatim or as near to the meaning literally described by the participant (Doody & Noonan, 2016). Study participants must be viewed as autonomous agents with the right to voluntarily accept or decline to participate in any study and to cease participation at any stage without prejudice. To uphold the principle of nonmaleficence, the researcher must pay close attention to the possible psychological consequences of participating in a study, particularly in qualitative research (Savin-Baden & Howell Major, 2013). According to Lowes and Gill (2006), interviews have the potential to evoke emotions and unexpected feelings. Therefore, preparation prior to data collection is advised to consider any potential consequence and arrange an appropriate referral system if required (Atkinson & Mannix McNamara, 2016) and should be integral in the research design.

Participants are susceptible to researchers imposing their own subjective interpretations that represent participant’s understandings (Danby & Farrell, 2004), although this is less of an issue in qualitative description design where the focus is primarily on rich description of the data and then on interpretation. Subjective interpretation raises issues of who owns the data, how will data be used, and how much control over the findings do participants have? Even though participants are given a voice, it is usually the researcher who decides on the direction that the research takes, the final interpretation of the data, and which information is reported. However, this does not contradict qualitative researchers’ focus on the veracity of the data; it is in fact fundamental to qualitative research to describe the individuals’ experiences. Researchers, therefore, have a responsibility to keep as near to the participants’ meaning as possible by using their own words and with a degree of interpretation that is consistent with the research question and the data collected.

### Data Collection

Data collection involves the use of data to understand and explain the phenomenon. The primary sources of data collection in qualitative description research are often semistructured in-depth interviews, but other methods are not discounted (Stanley, 2015). Data collection methods in qualitative description designs can include interviews, focus groups, observation, or document review (Colorafi & Evans, 2016). However, the use of interviews enables the researcher to explore issues with participants through encouraging depth and rigor, which facilitates emergence of new concepts/issues (Doody & Noonan, 2013; Fetterman, 1998) and contributes to the “richness of data” required in qualitative description designs.

According to Fetterman (1998), interviews take the researcher into the “heart of the phenomenon classifying and organising an individual’s perception of reality” (p. 40). Sandelowski (2000) suggests that a semistructured and open-ended interview guide be used to avoid limiting responses and to encourage participants to express themselves freely. Similarly, Sullivan-Bolyai et al. (2005) suggest the development or use of a framework to guide and focus interview questions, reflecting the relevant published literature as suggested by Miles, Huberman, and Saldana (2014). This framework may provide general or specific direction about topics to be addressed in interviews. Regardless of which template is used, it is important to ensure the focus remains on the original phenomenon of interest.

### Data Analysis

Qualitative data analysis predominantly consists of content or thematic analyses, which are often erroneously used interchangeably (Miles et al., 2014).There are many similarities in the above approaches including searching for patterns and themes (Vaismoradi, Turunen, & Bondas, 2013) and both can be used with good effect in the analysis of data from qualitative description studies. However, as noted by Vaismoradi et al. (2013), quantification of the data is more likely with content analysis which may fit better with the “straight description” of the data (Sandelowski, 2000) associated with qualitative descriptive designs. Nevertheless, use of a named framework for data analysis (Braun & Clarke, 2006; Burnard, 2011; Elo & Kyngas, 2008), which is carefully described, is vital to demonstrate the rigor of the study. Transcribing the interviews and listening to the voices of the participants repeatedly enables the transcriptions to come alive during the analysis in the quest for themes and subthemes, regardless of which framework for analysis is used. A large number of themes may be identified initially, but after further analysis and focusing on the purpose of the study, a smaller number of themes will stand out to capture the experience. These are described as “straight descriptions” of the data arranged in a way that “fits the data” (Sandelowski, 2000), a decision that can be verified by the participants through the member checks procedure (as a means of augmenting rigor) if agreed previously or desired. The various subthemes can then be captured by identifying similar or dissonant patterns within the themes. Data can be organized in tables to create a visual and contextual interpretation. However, although this process may appear linear, the analysis follows a circular movement and there may be several iterations made before establishing themes and subthemes emanating from the data. This repeated reading, reviewing, and refining of themes and subthemes while keeping in mind the whole text demonstrate how the iterative process includes comparisons on all types of data (Ayres, Kavanaugh, & Knafl, 2003). During this process, the researchers follow the data as concepts emerge, and stays open and close to what the data said and how it was said, creating an inductive process within the world of the data. Creswell (2014) calls this process “The Data Analysis Spiral.” Although emphasis is placed on description, analysis of qualitative description data by its very nature will involve some degree of interpretation (Sandelowski, 2010).

Adopting a flexible design such as qualitative description enables data collection and analysis to be an iterative process by responding to participant’s responses to questions and simultaneously adapting the analytical process as new insights emerge as the study progresses (Patterson & Morin, 2012). The advantage of a qualitative description approach is that data analysis is more likely to remain true to participants’ accounts and contribute to ensuring the researchers’ own interpretations are transparent (Clancy, 2013; Sandelowski, 2000).

### Rigor

The demonstration of quality regarding the research process and subsequently the data collected is essential for all approaches to research. However, qualitative research cannot be judged using the same criteria as the scientific paradigm. It is generally acknowledged that procedures to assess rigor within quantitative studies (validity and reliability) are inappropriate for qualitative research (Creswell, 2014). This does not suggest that qualitative researchers are unconcerned with data quality. It is in fact fundamental to qualitative research to demonstrate the truth of an individual’s experience and to ensure that the researcher presents a truthful representation of the participants’ voice and experience.

To demonstrate the quality of the data, qualitative researchers are concerned with issues of trustworthiness, which include principles of credibility, dependability, confirmability, and transferability. These principles were first introduced and developed in the 1980s by Lincoln and Guba (1985) to facilitate description of rigor within qualitative research. However, debate continues regarding the appropriateness or effectiveness of these concepts to demonstrate rigor in qualitative research. Morse, Barrett, Maynan, Olson, and Spiers (2002) are opponents of these concepts and argue that the terms *reliability* and *validity* remain the most appropriate criteria for attaining rigor in qualitative studies. These authors’ main criticisms are that the elements advocated to demonstrate trustworthiness are focused at the end of a study and are therefore evaluative in nature rather than identifiable or explicit during the research process. This, according to Morse et al. (2002), results in the continuing view that qualitative research is unscientific or less rigorous than quantitative research. However, Ryan-Nicholls and Will (2009) refute these claims. These authors stress the importance of acknowledging the epistemological positions of each research approach and argue the necessity of utilizing a process that best demonstrate rigor in qualitative research. Consequently, the four principles identified by Lincoln and Guba (1985) remain an important framework for all qualitative researchers to demonstrate the quality of their research and can be readily applied to qualitative description research. The authors of this article identify means to support these four criteria in Table 2 specific to qualitative description and note the importance of demonstrating rigor from the inception of the research and throughout the research process to address the concerns of Morse et al. (2002).

**Table 2.** Demonstrating Rigor in Qualitative Description Research.

Criteria	Means to Support
Credibility	● Established rapport prior to commencing interviews. ● Developing a trusting relationship (willingness to exchange information). ● Express compassion and empathy during interviews. ● Prolonged engagement. ● Participants to verify the accuracy of the interview transcript (member checking).
Confirmability	● Notes recorded in a reflective journal. ● An audit trail used to capture data collection and analysis process. ● Description of demographics of participants. ● Utilizing member-checking processes to verify data accuracy. ● Findings represent the data gathered and not biased by the researcher, evidenced by inclusion of direct quotations from participants.
Dependability	● Establishment of an audit trail describing the study’s procedures and processes. ● Account for any changes that occur within the study.
Transferability	● Purposeful sampling. ● Maintaining a reflexive journal. ● Providing sufficient study details so recreation could occur. ● Rich description.

*Source.* Developed by the authors.

Quality indicators for qualitative description research must reflect the philosophical underpinning of the research design and the research question. Finlay (2006) presents possible methods to engage in and demonstrate quality or trustworthiness within qualitative research. These include, for example, providing a detailed audit trail to defend decisions made during the research process, evidence of prolonged engagement with the narrative data and including the participants’ voice/narrative within the findings to demonstrate the quality of the research findings (Finlay, 2006). In addition, the practice of reflexivity is an essential component to incorporate into and engage within the research process to demonstrate trustworthiness (Finlay, 2006; Kingdon, 2005). Reflexivity is vital to augment the critical appraisal of the researcher in an analysis of the intersubjective dynamics between researcher and the participants. Reflexivity requires critical self-reflection of the ways in which researchers’ social background, assumptions, positioning, and behavior affect the research process (Finlay, 2006; McCabe & Holmes, 2009) which are often a factor when nurses and midwives are researching their practice areas. Therefore, the researcher is implicit in safeguarding the integrity of the study by demonstrating the study’s trustworthiness.

## Conclusion

Qualitative description research designs have been predominately used in nursing and midwifery research to provide direct descriptions of phenomena (Sandelowski, 2000). There is a clear alignment of qualitative description research with the philosophies and principles, which underpin both nursing and midwifery, including understanding and supporting the person, their family, and society as it explores meaning and/or how people make sense of the world and promoting person-centered/women-centered care. Qualitative description research provides a vehicle for the voices of those experiencing the phenomena of interest and can transform nursing and midwifery practice and indeed health care services generally by developing effective, culturally sensitive interventions, and make policy recommendations among those that are the focus of the research (Sullivan-Bolyai et al., 2005) and influence health care provision.

Qualitative description studies will have overtones of other qualitative methods, which is acceptable as noted by Law (2004). These overtones need to be acknowledged and described explicitly while recognizing that the research approach remains qualitative description and should be appropriately named (Sandelowski, 2000). A qualitative description approach needs to be the design of choice when a description of a phenomenon is desired, with a focus on the Who, What, Where, and Why of the experience (Neergaard et al., 2009). Researchers can confidently name their research design as qualitative description, and reference to description does not exclude the fact that an exercise of thought, practice of analysis, activity of reflection, and interpretation occurs.

This article provides the researcher with theoretical underpinning of a qualitative description approach, including the philosophical, ontological, and epistemological perspectives, which are the foundations of qualitative description research. In addition, key issues which are integral to the development of a research design, for example, methods, data collection, and data analysis are discussed in relation to qualitative description methodology. The key attributes and value of qualitative description research in the health care professions have been delineated with the aim of acting as a resource for researchers and extending the use of qualitative description in research.
